# Pessimistic expectations and poorer experiences: The role of (low) extraversion in anticipated and experienced enjoyment of social interaction

**DOI:** 10.1371/journal.pone.0199146

**Published:** 2018-07-05

**Authors:** Korrina A. Duffy, Erik G. Helzer, Rick H. Hoyle, Jun Fukukura Helzer, Tanya L. Chartrand

**Affiliations:** 1 Department of Psychology and Neuroscience, Duke University, Durham, North Carolina, United States of America; 2 The Johns Hopkins Carey Business School, Johns Hopkins University, Baltimore, Maryland, United States of America; 3 Merkle, Inc., Columbia, Maryland, United States of America; 4 Fuqua School of Business, Duke University, Durham, North Carolina, United States of America; Unviersity of Sheffield, UNITED KINGDOM

## Abstract

Given research suggesting that social interactions are beneficial, it is unclear why individuals lower in extraversion engage less in social interactions. In this study, we test whether individuals lower in extraversion reap fewer hedonic rewards from social interactions and explore social psychological processes that explain their experiences. Before participants socialized, we measured extraversion, state positive affect, cognitive capacity, and expectations about the social interactions. After participants socialized with one another, we measured state positive affect and cognitive capacity again as well as fear of negative evaluation and belief in limited cognitive capacity. Participants also rated the social skillfulness of each interaction partner. We found that less extraverted individuals expect to feel worse after socializing. However, all but those extremely low in extraversion (17% of sample) actually experience an increase in positive affect after socializing. Surprisingly, those low in extraversion did not show reduced cognitive capacity after socializing. Although they are more likely to believe that cognitive capacity is limited and to be fearful of negative evaluation, these characteristics did not explain the social experience of those low in extraversion.

## Introduction

As research accumulates on the defining features of trait extraversion, it is increasingly apparent that the stereotypic view of introverts (i.e., those low on the extraversion dimension) as uninterested in social interaction is, at best, overly simplistic, e.g., [1], and at worst, incorrect [2]. Although extraversion is positively associated with responsiveness and attentiveness to social stimuli [3], there is evidence that most individuals, regardless of their level of extraversion, report higher positive affect following social situations compared to non-social situations [4]. Despite this, individuals lower in extraversion are less likely to seek out social interaction for rewards such as positive affect and social attention [5]. This raises a puzzle: given that most people appear to benefit from social interactions, why do those lower in extraversion socialize less than those higher in extraversion [6],[7]? There may be many reasons. For example, it may be that they do not experience an increase in positive affect of the same magnitude as extraverts, that they have more negative expectations going into social interactions, or that they find social interactions require a greater amount of effort, which makes socializing more costly. We tested whether individual differences in extraversion are associated with different experiences before, during, or after social interactions, as well as the social factors that might undermine those experiences.

**Fig 1:**
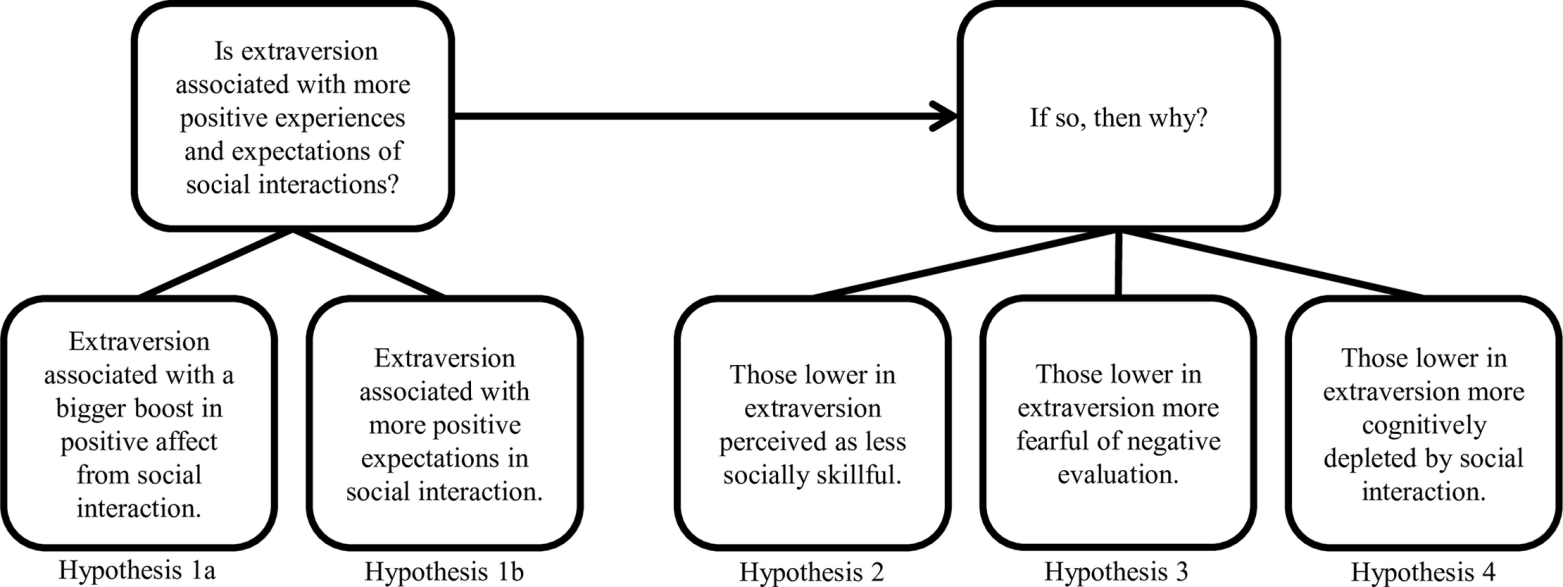
Conceptual diagram of research questions and the underlying hypotheses tested.

The purpose of our study was to examine two broad questions that lie at the intersection of personality and social psychology regarding the nature of extraversion and social interaction. The first question asks whether extraversion is positively associated with hedonic (i.e., affective) reward reaped from social interactions. Currently, evidence is mixed regarding the affective consequences of social interaction for those high and low in extraversion (see [8] and [9] for examples), so we examine both the expected and actual hedonic rewards resulting from interactions with strangers in a controlled setting. The second question (assuming an affirmative answer to the first question) asks why these experiences differ by extraversion. In particular, we focus on social-cognitive and affective processes that might help to explain these differing experiences. To clarify the specific hypotheses tested and how the paper is organized, see Fig 1.

### Lower extraversion and more negative affective experiences and expectations of social interactions

Positive affect has been shown to be both a motivator [10–12] and consequence [8],[13–16] of social interaction. In an early study showing that positive affect motivates people to socialize, positive or negative affect was induced and interest in social versus solitary activities was measured [11]. Participants induced to feel elated indicated greater interest in social events than participants induced to feel depressed, who indicated greater interest in solitary events. In a follow-up study of actual social behavior, participants who received a positive mood induction engaged in more sociable behavior and intimate self-disclosures than those who received a negative mood induction [10]. Thus, positive affect appears to be an important determinant of when people pursue social interaction.

With this in mind, it is noteworthy that for a long time extraversion has been associated with trait positive affect. Dating back to Eysenck, who argued that people with depression and anxiety disorders tended to be high in introversion [17], most theoretical accounts of extraversion include an affective component, such that more extraverted individuals experience higher levels of chronic positive affect [18],[19]. The fact that positive affect is implicated as a mechanism underlying both social interaction and trait extraversion focused us, first, on examining the affective expectations and experiences of individuals high and low in extraversion before, during, and after social interaction.

Affective responses are conceptualized as consisting of three components: valence (positivity/negativity), arousal (low to high), and motivational intensity (low to high). At a broad level, our first hypotheses concerned whether motivation to engage in social settings may be affected by both the anticipated and the actual valence and arousal associated with social interaction.

Social interactions have the potential to produce arousal that may be preferred or rewarding for individuals high, but not low, in extraversion. Because individuals may seek situations with optimal arousal, those high in extraversion may seek more stimulating situations [20–24]. Moreover, according to the affective-reactivity hypothesis, the same positive experience will create more positive affect for individuals higher versus lower in extraversion due to their stronger reactivity to rewards [25]. In their now classic paper, Larsen and Ketelaar showed across several studies that extraversion was associated with greater positive affect resulting from visualizing a range of life events [26].

Given that (a) as described earlier, most individuals experience positive affect from social situations; (b) extraversion is associated with greater positive affect for the same positive stimuli; and (c) social interactions may be optimally arousing for those high in extraversion, we hypothesized that most or all people would experience a boost in positive affect following social interaction, but that individuals higher in extraversion would experience this more (Hypothesis 1a). We designed the study this way in order to test whether extraversion moderates the effect of socializing on positive affect in a naturalistic setting. If those higher in extraversion experience a larger boost in positive affect following a social interaction, this would provide direct evidence that socializing provides a smaller boost in positive affect and is less beneficial for individuals lower in extraversion, setting the stage for Hypotheses 2–4 (see below), which explore why this is the case.

Regardless of actual change in positive affect following social interaction, extraversion may also be associated with differential expectations of enjoyment for the interaction. Expectancy effects are very commonly discussed in the psychological literature, and extant research suggests that expectations can shape one’s experiences [27–29]. In social situations, negative expectations about a social interaction can even increase the likelihood of being negatively evaluated [30].

Recent research suggests that, in general, people underestimate how good they will feel after socializing with others [9],[15],[31]. In two field studies, people mistakenly predicted that remaining in solitude while commuting on a bus or train would be a more positive experience than connecting with a stranger, when in actuality, people had a more positive commuting experience when they connected with a stranger [9]. Other research, though, suggests that extraversion should moderate these effects. In one study, for example, extraversion was positively associated with accurately predicting the benefits of acting extraverted [15]. Given these findings, we tested whether affective forecasts for social interactions vary with extraversion. We hypothesized that extraversion would be associated with more positive expectations for how they would feel following the social interaction (Hypothesis 1b).

Hypotheses 1a-1b offer direct tests of the affective-reactivity hypothesis; namely, whether those lower in extraversion actually experience less enjoyment or simply anticipate less enjoyment from social interaction. Hypotheses 2–4 probe the actual experiences of participants and their conversation partners in social settings to better understand how the experience of social interaction may differ based on individual differences in extraversion.

### Lower extraversion and less socially skillful behavior

An abundance of research has demonstrated that extraversion is positively associated with frequency of social interaction [32], a felt sense of community [33], positive perceptions of conversation partners [32], and perceived control in social contexts [32]. Individuals higher in extraversion create more favorable first impressions [34],[35], engender positive social environments [36], and elicit more liking from their social interaction partners [37]. Furthermore, research has shown that the ability to create more positive social experiences mediates the relationship between extraversion and positive affect [38]. Given these findings, as well as the predicted results of Hypotheses 1a and 1b, we postulated Hypothesis 2: individuals lower in extraversion would be perceived as less socially skillful by their interaction partners and being perceived as less socially skillful would be associated with lower positive affect post-social interaction. In order to examine this, we measured the impressions of multiple social interaction partners, unlike most previous research which has relied upon self-report [39–42] and third-party observers [32],[35].

### Lower extraversion and maladaptive cognitions

Extraversion is associated with attentional biases for valenced information: individuals high in extraversion tend to orient toward, and fixate upon, positive stimuli, whereas those low in extraversion tend to show biases for negative stimuli [43]. Moreover, in a social context, those low in extraversion are more likely to recall information about opponents versus teammates whereas the reverse is true of those high in extraversion [44]. This negativity bias may result in greater salience of potential threat in social encounters for those low in extraversion. Their past aversive social encounters might be particularly salient when anticipating future social encounters, which would prompt greater anxiety about these situations. Because of this negativity bias, we postulated Hypothesis 3: extraversion would be negatively associated with fear of negative evaluation. Furthermore, because research shows that negative expectations about a social interaction can increase the likelihood of actually being socially rejected [30], we hypothesized that increased fear would result in less positive evaluations by conversation partners.

Previous research has shown a link between socializing and cognitive functioning. Depending on the context, socializing can boost cognitive functioning [45],[46] or impair it [47–50]. In a laboratory study, engaging in a ten-minute social interaction improves processing speed and working memory performance [45]. Furthermore, social interactions that are cooperative boost cognitive performance [46]. However, social interactions that occur in certain contexts do not come with these benefits. For example, if the social interaction is competitive rather than cooperative, then cognitive performance is not improved [46]. Some social interactions are even cognitively depleting, such as those that are high maintenance, characterized by a lack of social coordination [47]. Furthermore, interacting with someone of a different race can also impair cognitive function if one scores high on prejudice towards a racial outgroup [50] or favoritism towards a racial ingroup [49]. In these studies, reduced cognitive function was indicated by slower reaction times and less accurate performance on the color-naming version of the Stroop Test. Thus, social interactions seem to impair cognitive function in contexts in which social interactions are effortful. Despite the fact that research has investigated the contexts that moderate the effect of social interactions on cognitive function, little research has explored individual differences that may moderate these effects.

Extraversion may moderate the effect of social interaction on cognitive function. In particular, social interactions may be differentially exhausting for those low in extraversion because less effective social coordination is more effortful [51] and avoidance motivation is more taxing (because it involves heightened vigilance, greater attention to detail, and increased recruitment of cognitive resources [52]). Despite self-report evidence that introverts feel a reduction in cognitive capacity after socializing (i.e., in line with Jung’s original conception of introverts and extraverts, which distinguished between them on the basis that introverts are exhausted by social interaction while extraverts are energized by it), no research has empirically tested this in a naturalistic context (i.e. unscripted, free form social interactions). Zelenski, Santoro, & Whelan [16] tested this in a non-naturalistic context, in which participants were randomized into one of three conditions: acting introverted, acting extraverted, or no acting instructions (control condition). In groups of three with each participant assigned to a different acting condition, participants interacted on a given discussion topic (i.e. describing the usefulness of 10 items following a winter plane crash) and then Stroop performance was measured. They found that acting introverted led to greater depletion compared to the control group, but only for those higher in extraversion. However, this paradigm did not capture the consequences of social interaction in a naturalistic setting. Furthermore, this study did not mention whether dispositional extraversion predicted Stroop performance in the control condition (with no acting instructions), likely because the sample size was too low to parse the data in this way (117 participants were divided between 3 conditions).

Thus, to the extent that extraversion is associated with more socially skillful behaviors (Hypothesis 2) and less avoidance and fear of social interaction (Hypothesis 3), the experience of social interaction may be more effortful and, thus, more depleting for those low in extraversion. If socializing is more effortful for individuals lower in extraversion, this should lead to a reduction in cognitive capacity following an interaction with strangers. In the current study, we measure Stroop Test performance before and after socializing in order to test Hypothesis 4.

In addition, recent research has demonstrated that individuals differ in their belief that cognitive capacity is limited (i.e., how “depletable” they are) and that these beliefs moderate actual depletion following an effortful task [53]. Thus, to fully test Hypothesis 4, we also measured beliefs in cognitive capacity to examine whether individuals high versus low in extraversion differ in their beliefs that cognitive capacity is limited (i.e., “depletable”). We examined this question in its own right and then tested whether such beliefs were related to actual depletion and/or affective costs.

## Methods

### Study design

We tested these research questions in a controlled context that simulated real-life social interactions at zero-acquaintance. The context we employed was patterned after a cocktail party–participants were given 20 minutes to interact in free form with 3 to 10 strangers. Participants were instructed to try to talk with everyone and strongly encouraged to talk in dyads. They were, however, not told what to talk about. Before and after the social interaction, all participants were surveyed regarding their expectations (pre-social interaction) and actual experiences (post-social interaction) in the “cocktail party.”

### Participants

One hundred fifty-five participants (ages 18–30; 70% female) were recruited from a university participant pool for a 60-minute study and were compensated $15. All participants provided written informed consent. This study received approval from the Duke University Institutional Review Board (Protocol B0918) and complied with the principles expressed in the Declaration of Helsinki.

We were concerned that a self-selection bias would emerge if participants knew that they would be engaging in social interactions when they signed up for the study, so participants were not informed about the social interaction component of the study until they arrived. Participants completed the study in groups of 4 to 11 for a total of 22 groups. For one group of ten participants, there was a technological problem five minutes into the study. These participants were compensated $5 for their time and allowed to sign up again. Three of these participants completed the study in a different session. In addition to the seven participants who were excluded due to technological problems, two participants were excluded due to missing questionnaire data. After excluding these nine participants, we had a total sample of 146 participants.

Similar studies report medium and large effect sizes [9],[13],[15],[19]. Based on these effect sizes, we did a power analysis to estimate sample size based on *r* = .3 for medium effect sizes. Sample size was estimated at 85. In order to be able to detect smaller effects (e.g., moderated effects), we aimed for a sample size of 150. The data for this study have been made publically available on Open Science Framework (osf.io/98twx).

### Before arriving at lab

#### Measure of extraversion

Before coming to the lab, participants filled out the 60-item version of the HEXACO-PI-R [18]. We were interested only in the extraversion dimension and its facets (10 items; 1 = *strongly disagree*, 2 = *disagree*, 3 = *neither agree nor disagree*, 4 = *agree*, 5 = *strongly agree*); however, we administered the complete measure so that participants would not realize that we were interested in extraversion. From these 10 items, we created a composite score of extraversion (alpha = .80). In our sample, extraversion scores ranged from 1.8–5.0.

### Pre-social interaction measures

When participants arrived at the lab, they were led into a large testing room with multiple computers. Participants were seated in front of one of the computers and instructed to complete the pre-social interaction questionnaire, which consisted of the following measures:

#### Pre-social interaction positive affect

Recent research has demonstrated that the model of postive affect that is most closely associated with extraversion taps into the positive valence and high activation components of positive affect [54]. As a measure of each of these, participants reported on a 7-point scale how they felt at the moment (upon arriving to the study) both in terms of valence (*very unhappy* to *very happy*) and activation (*very exhausted* to *very energized*). These scores (*r* = .42, *p* < .001) were added together to create a composite score of *pre-social interaction positive affect* (also referred to as baseline). In our sample, composite scores ranged from 4–14. Importantly, this was measured upon arrival to the study before participants even knew that they would be engaging in a social interaction with the other participants in the study.

#### Pre-social interaction cognitive capacity

In order to measure cognitive capacity before the social interaction, participants completed the Stroop Test. Participants were told that they would be presented with a screen on which was either a *color word* (BLUE or RED) or *empty symbols* (XXXX). The color of the text was either blue or red. Participants were instructed to indicate the color of the word as quickly as they could and ignore the meaning of the word. After completing 4 training trials, subjects completed 100 trials total– 40 trials were congruent, meaning the color word matched the text color (i.e. the word BLUE written in blue text), 40 trials were incongruent, meaning the color word did not match the text color (i.e. the word BLUE written in red text), and 20 trials involved empty symbols that were either in red or blue font. Using the keyboard, participants were instructed to press ‘B’ on the keyboard if the word was written in blue text and ‘R’ if the word was written in red text. Reaction times were measured by Qualtrics, the computer program used during the experiment.

#### Anticipated positive affect

Participants were then told for the first time that they would be engaging in a social interaction with the other participants in the room. They were given the following information:

“In the next portion of the study, you will have 20 minutes to try to get to know the other participants in the room. Think of this as a cocktail party in which everyone mingles with each other. You are encouraged to try to spend your time getting to know everyone. First, though, you will answer a series of questions about how you anticipate feeling during and after the social interaction.”

After reading this, participants were asked to predict their state positive affect after the social interaction using the same scales as the pretest measure to assess how they anticipated they would feel both in terms of valence (*very unhappy* to *very happy*) and activation (*very exhausted* to *very energized*). The scores on these two items (*r* = .52, *p* < .001) were added together to create a composite score of *anticipated post-social interaction positive affect*. In our sample, composite scores ranged from 3–14.

### Social interaction

After participants had completed the pre-social interaction questionnaire, they engaged in a 20-minute social interaction with the other participants in the session. In order to enable the collection of data on each of the social interactions, participants were told to try to get to know everyone equally. They were informed that they would be asked about each of the other participants afterward. In order to encourage active participation in each interaction, participants were told that they should try to interact in dyads, although groups with three people were also acceptable. Participants were told how much time they had remaining every five minutes so that they could allocate their time appropriately in getting to know all of the other participants.

#### Post-social interaction measures

After the 20-minute social interaction, participants completed the post-social interaction questionnaire, which consisted of the following measures:

#### Post-social interaction positive affect

In order to measure state positive affect after the social interaction, we asked participants to report again how they felt at the moment both in terms of valence (*very unhappy* to *very happy*) and activation (*very exhausted* to *very energized*). The scores on these two items (*r* = .74, *p* < .001) were added together to create a composite score of *post-social interaction positive affect*. In our sample, composite scores ranged from 2–14.

#### Post-social interaction cognitive capacity

In order to measure cognitive capacity after the social interaction, participants completed the Stroop Test again. By measuring performance on the Stroop Test before and after the social interaction, it was possible to measure change in cognitive capacity. In order to calculate reaction times, incorrect trials were removed and then means were calculated for the pre- and post-social interaction Stroop Tests. We excluded seven participants with an average reaction time +/- three standard deviations from the group mean on either the pre- or post-Stroop Test based on standard procedure with Stroop data [55],[56].

#### Social skillfulness (rated by partners)

Social interaction partners rated target participants on five 7-point scales. Social interaction partners only rated participants that they did not know before coming to the study. Therefore, each participant had reports from each of their social interaction partners that they did not previously know (more than 95% of the time, participants were not previously acquainted).

Two items assessed characteristics of the target participant as assessed by their social interaction partners. These items asked social interaction partners how introverted or extraverted (*very introverted* to *very extraverted*) and how socially awkward or socially skillful (*very socially awkward* to *very socially skillful*) they perceived the target participant to be. One item assessed how effortful or effortless it was to interact with the target participant (*very effortful* to *very effortless*). Two items assessed liking; one for how much the social interaction partner disliked or liked the target participant (*disliked* to *liked*) and one for how much the social interaction partner felt disliked or liked by the target participant (*disliked* to *liked*). The scores for each item were averaged for each target participant across all the social interaction partners. Given that the responses on the five items hung together closely (alpha = .92) and all seemed to tap into how socially skillful the target participant was perceived, the responses on the five items were averaged to create a composite of *social skillfulness*. In our sample, composite scores ranged from 2.8–6.4.

#### Belief in limited cognitive capacity

Participants completed the Implicit Theories About Willpower Scale [53] to measure theories about the effects of mental exertion. For all six items, participants indicated the extent to which they agreed with the statement (*strongly disagree*, *moderately disagree*, *slightly disagree*, *slightly agree*, *moderately agree*, to *strongly agree*). Those with a higher belief in *limited* cognitive capacity tended to agree with statements such as: “After a strenuous mental activity, your energy is depleted and you must rest to get it refueled again.” Those with a higher belief in *non-limited* cognitive capacity tended to agree with statements such as: “Your mental stamina fuels itself. Even after strenuous mental exertion, you can continue doing more of it.” The three items that endorsed belief in non-limited cognitive capacity were reverse scored and then all six items (alpha = .88) were averaged to calculate a score for each participant called *belief in limited cognitive capacity*. In our sample, composite scores ranged from 1–6.

#### Fear of negative evaluation

Participants completed the 12-item version of the Fear of Negative Evaluation Scale [57], used to measure the degree to which people experience apprehension at the prospect of being evaluated negatively. For each statement, i.e. “I am afraid that people will not approve of me,” participants indicated how characteristic the statement was of them (*not at all*, *slightly*, *moderately*, *very*, and *extremely*). Four items were reverse scored and then all twelve items (alpha = .91) were averaged to create a composite score for each participant. In our sample, composite scores ranged from 1–5.

For descriptive statistics of all variables and intercorrelations between variables, see Table 1.

**Table 1:** Means (*standard deviations*) and intercorrelation matrix of measured variables.

		M (*SD*)	1	2	3	4	5	6	7	8	9	10	11
1	Extraversion	3.44 *(0*.*58)*											
2	Fear of Negative Evaluation	3.12 *(0*.*80)*	-.32										
3	Theories of Depletion	3.09 *(1*.*02)*	-.27	.27									
4	Pre-PA	8.97 *(1*.*83)*	.17	-.17	-.25								
5	Post-PA	10.20 *(2*.*24)*	.43	-.14	-.20	.51							
6	Anticipated-PA	8.55 *(2*.*02)*	.40	-.24	-.19	.39	.54						
7	Change in PA	1.23 *(2*.*05)*	.32	.002	.008	-.34	.64	.24					
8	Predicted Change in PA	-0.42 *(2*.*14)*	.23	-.08	.03	-.49	.08	.61	.52				
9	Perceived Social Skillfulness	4.91 *(0*.*07)*	.34	.06	-.03	.13	.32	.22	.23	.10			
10	Pre-Stroop Test (RT)	0.63 *(0*.*18)*	-.03	-.02	-.04	.05	-.04	.12	-.08	.08	-.005		
11	Post-Stroop Test (RT)	0.62 *(0*.*17)*	-.06	.04	-.13	-.002	-.06	.04	-.06	.04	-.02	.72	
12	Change in Stroop Test (RT)	-0.01 *(0*.*13)*	-.04	.08	-.12	-.06	-.03	-.11	.03	-.05	-.02	-.39	.36

For variables 1–9, *N* = 146 (*r* > .23, *p* < .05; *r* > .27 , *p* < .01). For variables 10–12, *N* = 139 (*r* > .24, *p* < .05; *r* > .29, *p* < .01).

## Results

### Analytic plan

The inferences we sought from our data were at the level of the individual rather than the group. However, given that individuals participated in the study within groups, we first needed to determine whether group characteristics accounted for significant variance in our dependent variables, specifically those that could have been affected by the social interactions (i.e. change in positive affect, perceived social skillfulness, and change in Stroop Test performance). Using MLM, we estimated group effects. Despite the downward biasing of standard errors at Level 2 when the number of groups is small (i.e., less than 50) [58], in the unconditional models, there were no significant effects of group on our dependent variables. Therefore, for ease of interpretation and because we were interested in individual-level inferences and wanted to do follow-up analyses not available in MLM, we presented our results using OLS rather than MLM. However, when we tested our effects accounting for group variance using MLM, all of our results held.

### Test of Hypothesis 1a: Extraversion is associated with a bigger boost in positive affect from social interaction

In order to test whether change in positive affect after socializing differs by extraversion, we first created difference scores by subtracting pre-social interaction positive affect from post-social interaction positive affect. This difference score represents the actual change participants experienced in their affective experience following the social interaction. Then, we ran a simple linear regression analysis by regressing change in positive affect on trait extraversion. The resulting model was significant, *R*^*2*^ = .10, *F*(1, 145) = 16.54, *p* < .001, indicating that individuals lower in extraversion experienced less change in positive affect after socializing, *B* = 1.13, *t*(144) = 4.07, *p* < .001.

**Fig 2:**
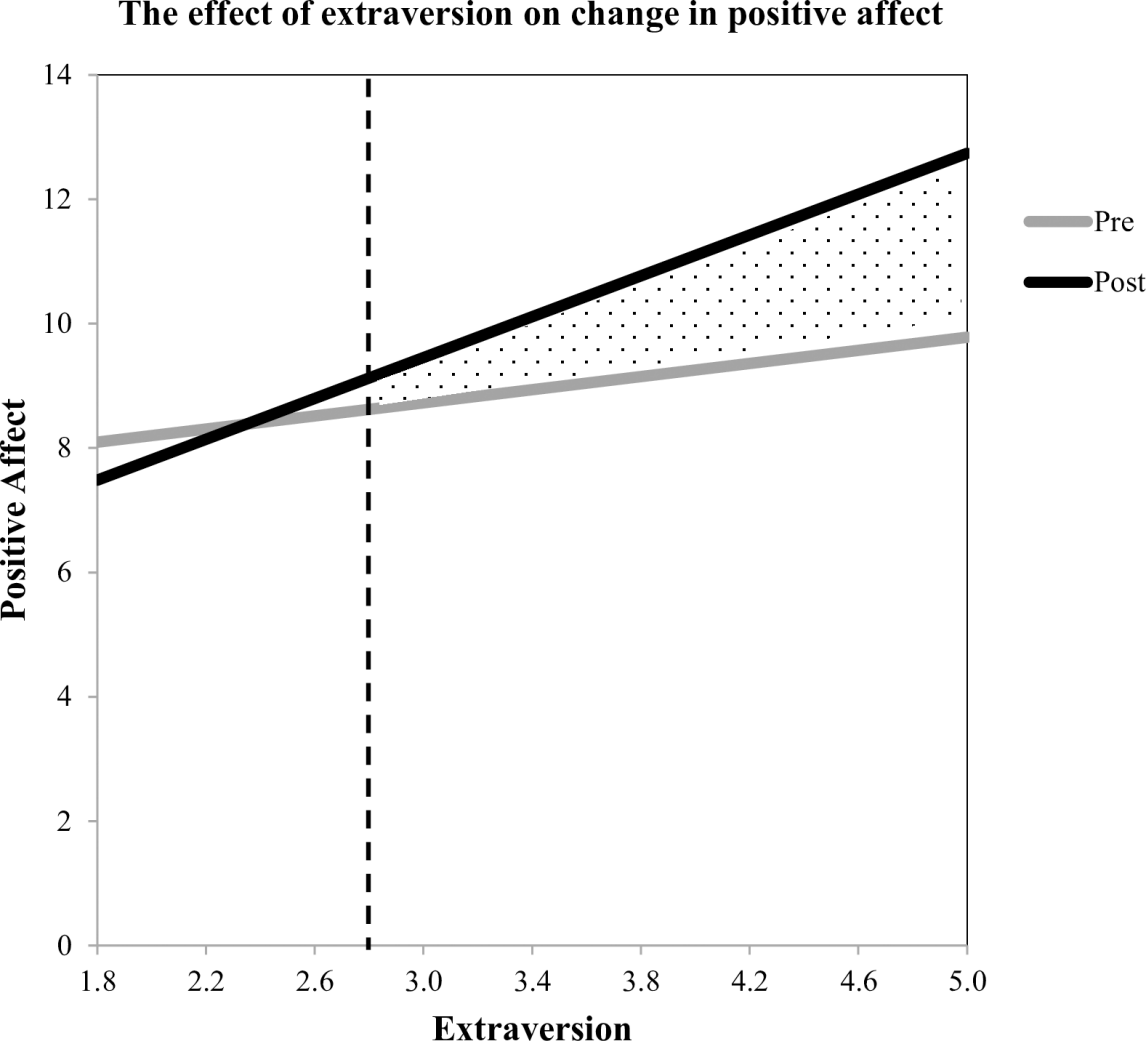
The relationship between extraversion and positive affect before and after socializing. A spotlight analysis revealed a region of significance indicated graphically by the gray zone. Participants who are more introverted (< 2.78, 17% of sample) did not experience a boost in positive affect. However, participants who are more extraverted (> 2.78, 83% of sample) experienced statistically significant increases in positive affect after socializing with others.

Given that the interaction effect was significant, we conducted a spotlight analysis in order to determine the level of extraversion at which change in positive affect was significant (the region of significance) [59]. We found that post-social interaction increases in positive affect were significant once extraversion levels exceeded a score of 2.78 (see Fig 2). To put this in perspective, 83% of our sample scored above this level on extraversion, and based on norming data (available at HEXACO.org), it is estimated that 88.1% of people (based on a Z-score of -1.18) in the general population will score above this threshold on extraversion. This suggests two things that are obscured by the results of the linear regression: first, the vast majority of participants, including those who score below the sample mean on extraversion (*M* = 3.53 for the sample, *M* = 3.51 for the population, HEXACO.org), experienced a boost in positive affect following social interaction; second, those who were exceptionally low in extraversion (the bottom 17% of the sample) experienced neither an increase nor a decrease in their positive affect after a 20-minute interaction.

### Test of Hypothesis 1b: Extraversion is associated with more positive expectations for the social interaction

In the next analysis, we tested whether extraversion was associated with more positive expectations for the social interaction, specifically, whether those high in extraversion expect a greater affective “boost” from social interaction than those low in extraversion. In order to test this, we created difference scores by subtracting pre-social interaction positive affect from anticipated post-social interaction positive affect and ran a simple linear regression by regressing the difference score on trait extraversion. The model was significant, *R*^2^ = .05, *F*(1, 144) = 8.01, *p* = .005, indicating that extraversion was associated with anticipated change in positive affect, *B* = 0.84, *t*(144) = 2.83, *p* = .005, *SE* = 0.30.

**Fig 3:**
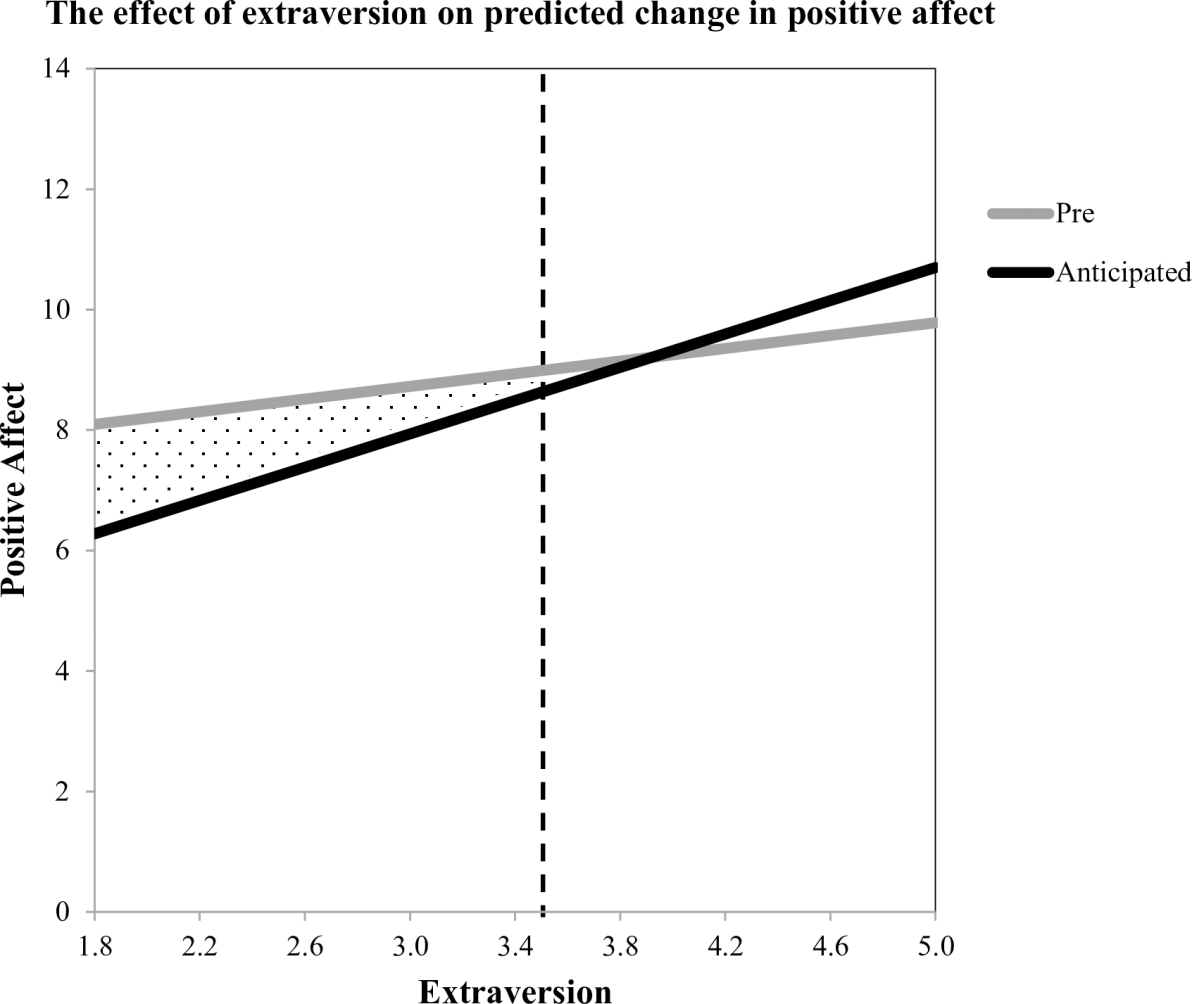
The relationship between (1) extraversion and baseline positive affect and (2) extraversion and what participants predicted their positive affect would be after socializing. The gray zone shows the region in which anticipated positive affect is significantly different from pre-positive affect. Participants who are more introverted (< 3.53, 55% of sample; *M* = 3.51 for population) expected to experience a decrease in positive affect after socializing with others. Those who are more extraverted did not predict a significant change in positive affect.

As shown in Fig 3, a spotlight analysis indicates that participants scoring lower than 3.53 on extraversion expect their level of positive affect to decrease from baseline to post-social interaction. Looking simultaneously at Figs 2 and 3, it is interesting to note the pattern for participants scoring between 2.78 and 3.53 on extraversion (38% of our sample). These individuals, who are at or below the population mean of extraversion (*M* = 3.51, HEXACO.org), anticipate taking an affective hit from social interaction, but end up receiving a boost instead. This suggests that individuals below the mean on extraversion (who might be self-described introverts) may harbor unduly pessimistic expectations for social interaction: they expect to feel worse, even though they feel better.

### Test of Hypothesis 2: Extraversion predicts being perceived as more socially skillful and this is associated with greater increases in positive affect after socializing

In order to test whether individuals lower in extraversion were perceived as less socially skillful, we correlated trait extraversion scores with ratings of how socially skillful the participant was perceived by social interaction partners. Indeed, extraversion positively predicted ratings of social skillfulness, *r* = 0.34, *p* < .001. In a follow-up analysis, we found that partner ratings of social skillfulness were positively correlated with participants’ change in positive affect, *r* = .23, *p* = .005, such that those who were perceived as more socially skillful also benefitted from greater increases in positive affect from baseline.

### Test of Hypothesis 3: Extraversion is negatively associated with fear of negative evaluation, but fear may be adaptive

As predicted, we found that extraversion and fear of negative evaluation were significantly and negatively correlated, *r* = -0.32, *p* < .001. We had hypothesized that, for introverts, this fear would contribute to poorer performance in the interaction. In order to test this, we began by estimating a moderated multiple regression model in which perceived social skillfulness (as rated by interaction partners) was predicted by trait extraversion, fear of negative evaluation, and their interaction. The model accounted for a significant proportion of variance in social skillfulness ratings, *R*^2^ = .17, *F*(3, 142) = 10.01, *p* < .001. Consistent with correlational results presented earlier, extraversion, *B* = 1.26, *t*(142) = 3.38, *p* = .001, *SE* = 0.37, and fear of negative evaluation, *B* = 0.93, *t*(142) = 2.56, *p* = .01, *SE* = 0.36, were significant predictors. Our primary interest was the interaction effect, which was also significant, *B* = -0.23, *t*(142) = -2.16, *p* = .03, *SE* = 0.11. (When we ran our main analyses controlling for group size, all effects held except the interaction effect of extraversion by fear of negative evaluation on perceived social skillfulness, which became marginal, *B* = -0.20, *t*(141) = -1.86, *p* = .065, *SE* = 0.11).

**Fig 4:**
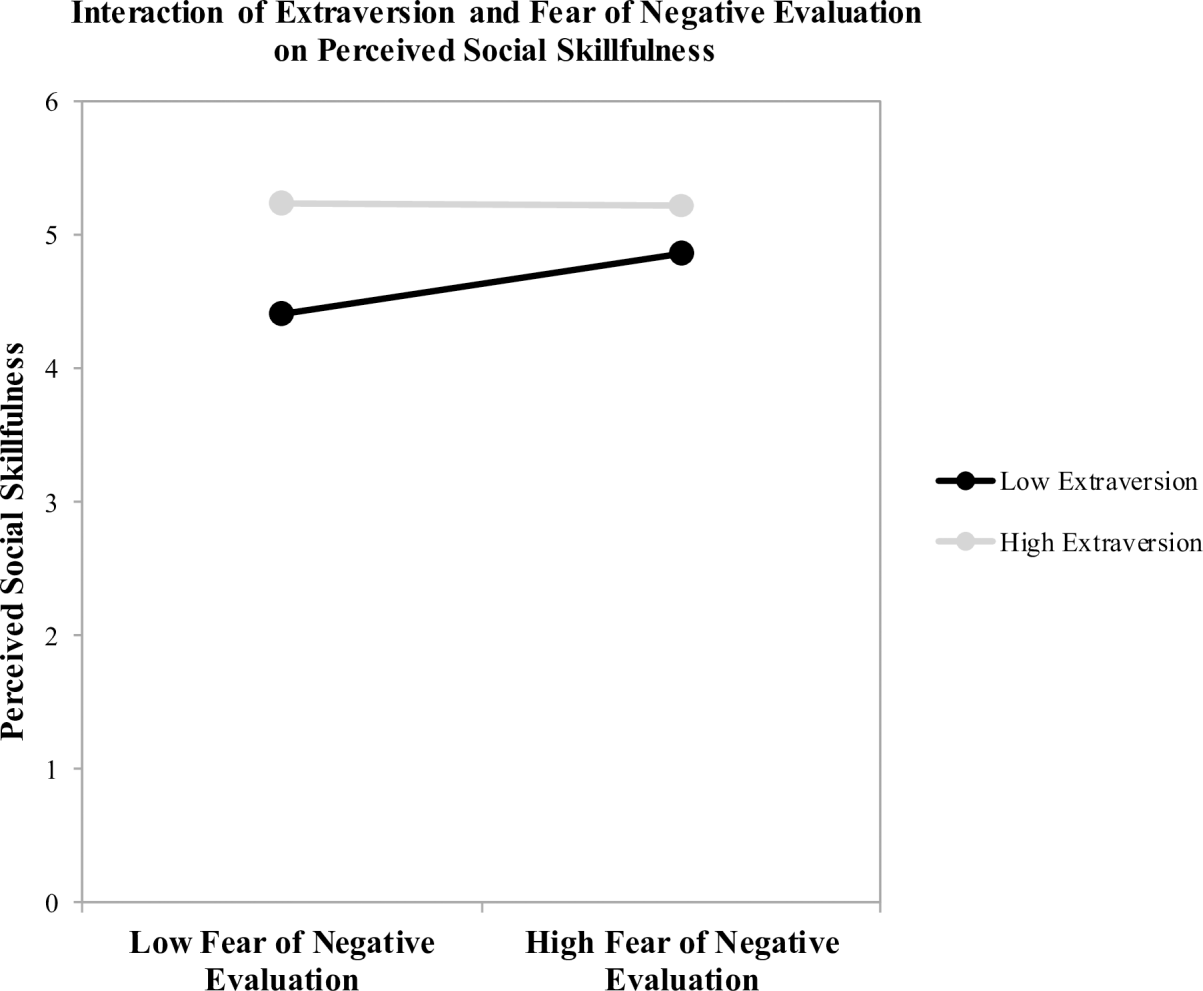
The interaction of extraversion and fear of negative evaluation on perceived social skillfulness. Plotted at 1 standard deviation +/- the mean for extraversion. Introverts higher in fear of negative evaluation were rated as more socially skillful by their interaction partners than introverts lower in fear of negative evaluation. Fear of negative evaluation did not predict differences in social skillfulness for extraverts.

As can be seen in Fig 4, the interaction revealed a surprising pattern. For those 1 SD above the mean in extraversion, fear of negative evaluation did not predict perceived social skillfulness. However, for those 1 SD below the mean, fear of negative evaluation was positively, rather than negatively, associated with perceived social skillfulness: introverts higher in fear of negative evaluation were rated as more socially skillful by their interaction partners than introverts lower in fear of negative evaluation. This suggests that fear of negative evaluation may motivate introverts to compensate for the suite of psychological processes that diminish their performance in social interactions.

### Test of Hypothesis 4: Extraversion does not predict a greater reduction in cognitive capacity after socializing, but is associated with beliefs about limited cognitive capacity

In order to test whether changes in cognitive capacity following social interaction were associated with extraversion, we first computed change in Stroop Test reaction time by subtracting pre-Stroop Test reaction time from post-Stroop Test reaction time. Next, we ran a simple linear regression analysis predicting change in Stroop Test performance from trait extraversion. The resulting model, however, was not significant, *R*^2^ = .001, *F*(1, 137) = 0.16, *p* = .69. (The model is also non-significant when the analysis is run with no participants excluded on the Stroop Test, *R*^2^ = .01, *F*(1, 144) = 1.41, *p* = .24). Thus, we found no evidence that extraversion is associated with actual cognitive depletion following social interaction.

Although there were no observable effects on actual depletion, individual differences in extraversion may be associated with different beliefs about how “depletable” one is–i.e., those lower in extraversion may feel that their cognitive resources require more “recharging” following social exertion. Indeed, participants who were less extraverted ascribed to a limited resource theory, *r* = -0.27, *p* = .001. When we tested whether belief in limited cognitive capacity is correlated with change in cognitive capacity and change in positive affect, both correlations were non-significant, *r*s = .008 and .08, respectively, *p*s = .93 and .37, respectively. These results, combined with those reported immediately above, suggest that while those lower in extraversion believe themselves to be more depletable, they show no signs of being more depleted than those higher in extraversion following social interaction.

## General discussion

This research addressed two questions: is extraversion associated with different hedonic rewards reaped from social interactions, and if so, what social psychological processes might explain these differing experiences? In order to test these research questions, we designed a study with a naturalistic paradigm, which allowed participants to engage in free form conversations with multiple other participants, simulating what would happen in actual social gatherings.

In addressing the first overarching research question, we show that extraversion is associated both with expected and actual changes in affective experience following social interaction. Although the overwhelming majority of our participants experienced a hedonic boost from this brief social interaction, individuals higher in extraversion reaped greater hedonic rewards than those lower in extraversion. Although previous research shows that, in general, socializing increases positive affect [60],[61], to our knowledge, no research has shown that extraversion moderates this relationship, and this finding might help to explain why individuals lower in extraversion may be less motivated to socialize. Although it can be difficult to determine the causal direction (i.e. whether diminished hedonic experience leads to lesser willingness to socialize, or vice versa), it is clear that experiences of the social interaction differed based on extraversion.

Of additional interest is how these patterns of actual change in positive affect corresponded (or not) with participants’ expectations of how they thought they would feel following the social interaction. Consistent with a tendency for those lower in extraversion to show negativity biases in social cognition, e.g. [43], the most introverted 17% of our sample expected a decrease in positive affect after socializing, but actually experienced no change; while extraverted participants (45% of sample who were above the population mean on extraversion) predicted no change in positive affect but actually experienced the largest increases. The remaining participants who were at or below the population mean on extraversion experienced a boost in positive affect following social interaction but anticipated a negative change. This suggests that despite thousands of experiences in social interactions, participants’ intuitions about how social interaction affects them are not particularly well calibrated. As others have pointed out [9], this may contribute to people missing out on hedonically pleasing experiences (regardless of their extraversion level) because they fail to anticipate their reward value.

To our knowledge, our results are the first to show that extraversion moderates the relationship between social interaction and changes in positive affect. Research supporting the affective-reactivity hypothesis has demonstrated that people higher in extraversion experience increases in positive affect more than people lower in extraversion during situations in which rewards are being pursued. In situations that are merely pleasant (but non-reward seeking), people high and low on extraversion respond similarly [62]. In line with this work, the fact that extraversion predicts greater increases in positive affect after socializing suggests that social interactions are inherently rewarding. In fact, neuroscience research shows that reward-related brain regions are activated during many aspects of social interaction, e.g. anticipating positive feedback, viewing beautiful faces as well as facial expressions of positive emotions, using theory of mind [63]. Thus, our study finds support for the affective-reactivity hypothesis using a context that is particularly important for the study of extraversion: actual social interactions.

Two papers have been published that are similar in some respects to our own. Below, we summarize these papers briefly and then discuss them in the context of our own findings. Unlike our study, which investigates the experiences of those naturally high and low in extraversion within a naturalistic social interaction, these papers have explored the effect of “acting extraverted” on positive affect. In these studies, extraverted and introverted behavior is manipulated by assigning participants either to act extraverted (bold, talkative, energetic, active, assertive, and adventurous) or to act introverted (reserved, quiet, lethargic, passive, compliant, and unadventurous) before engaging in a social interaction [15],[16]. Two studies tested whether introverts suffer a cognitive cost of acting counterdispositionally (i.e. acting extraverted) by measuring the cognitive and emotional costs and benefits of acting extraverted and introverted [16]. Regardless of disposition, acting extraverted was associated with higher positive affect than acting introverted and only extraverts acting introverted suffered a cognitive cost [16]. However, this finding led to a conundrum: if introverts do not seem to suffer a cognitive cost from acting extraverted and seem to benefit affectively from doing so, then why do they not act extraverted more often? This research question led to a second set of studies focused on whether introverts make affective forecasting errors that cause them to underpredict the benefits of acting extraverted [15]. In general, these studies showed that when assigned to act extraverted, introverts overestimated their negative affect and self-consciousness more so than extraverts, who were more accurate in their predictions. Therefore, the authors conclude that this may explain why introverts do not engage in extraverted behavior more often even though they may benefit from it.

Our study focuses on many of the same underlying research questions. However, we use the theoretical foundation of these experimental studies to expand on these questions in a naturalistic context. In particular, we considered it critical to assess actual change in positive affect by measuring baseline positive affect (pre-social interaction) as well as post-social interaction positive affect. Although these previous studies show that introverts seem to benefit from acting extraverted based on their post-social interaction positive affect relative to their post-social interaction positive affect when acting introverted, these studies did not measure baseline affect and therefore could not address whether introverts and extraverts benefit equally from social interactions, particularly as they occur naturally (i.e. no acting instructions).

Although it can be difficult to extrapolate how the effects of “acting extraverted” relate to experiences in real-life, some of these studies did include a control condition with no acting instructions. As a manipulation check, participants reported to what extent state extraversion items described how they acted during the social interaction. The results for the control were more similar to the acting extraverted condition than the acting introverted condition, suggesting that the default in social interactions is to act extraverted. This would explain the congruence between previous research and our study: whether participants are acting extraverted or needing to behave more extraverted due to the demands of socializing, introverts seems to have more negative expectations and worse experiences in social interactions.

In addressing the second overarching research question, we sought to understand the social psychological processes that might underlie differing social experiences for individuals across the extraversion continuum. The first process we tested was whether increases in positive affect after socializing could be explained by how successful the social interactions were. Unlike most previous research that has relied on self-report and third-party observers, we measured how participants were perceived by multiple social interaction partners. Although we did not measure specific behaviors, we show that individuals lower in extraversion were rated as less socially skillful and that being perceived as less socially skillful is associated with less affective benefits of the social interaction. Because conversations with less socially-skillful individuals involved more effort, greater awkwardness, and less liking (items that compose our social skillfulness index), the experiences of both social interaction partners were likely diminished. However, we find this to be particularly true for the less socially skillful person who has less of an affective boost from the social interactions.

The second process we tested was whether those lower in extraversion who are also more fearful of negative evaluation perform more poorly in social interactions. To our surprise, this fear worked in a way that contradicted our hypothesis. For individuals lower in extraversion, those higher in fear of negative evaluation were actually perceived by their partners as more socially skillful than those lower in fear of negative evaluation. Therefore, among those who approach interaction opportunities less frequently or with less enthusiasm, it seems that fear of negative evaluation may act as a motivator that leads to success rather than self-defeating trait that leads to failure. This finding is in line with previous research that shows that those high in fear of negative evaluation are more concerned with making good impressions and try harder to make a good impression during face-to-face interactions [64]. It is possible that these fears actually allow those lower in extraversion to compensate for other psychological processes that lead them to generally make less favorable impressions in social interactions.

The third process we tested was whether individuals lower in extraversion were more depleted after socializing. Despite self-report evidence that introverts feel a reduction in cognitive capacity after socializing, no research had empirically tested this in a naturalistic context. In our study, we tested this hypothesis with a naturalistic paradigm in which everyone was free to behave in line with their natural inclinations. We did not find that individuals lower in extraversion were particularly cognitively depleted. Extraversion was not associated with changes in cognitive capacity following social interaction, and, although individuals lower in extraversion were more likely to believe that cognitive capacity is a limited resource, this belief does not translate into reductions in cognitive capacity or positive affect following social interaction. (For a summary of our main findings, see Table 2).

**Table 2:** Main findings for each hypothesis.

Main Findings	
1a	Extraversion is associated with a bigger boost in positive affect from social interaction.
1b	Extraversion is associated with more positive expectations for the social interaction.
2	Extraversion predicts being perceived as more socially skillful and this is associated with greater increases in positive affect after socializing.
3	Extraversion is negatively associated with fear of negative evaluation, but this fear may be motivating.
4	Extraversion does not predict a greater reduction in cognitive capacity after socializing, but is associated with beliefs about limited cognitive capacity.

### Limitations

Although our study provides novel insights into why experiences in social interactions differ across the extraversion continuum, our study has limitations that should be considered when interpreting our findings. One consideration is that the Stroop Test may not adequately measure the phenomenon we intended to study. We chose the Stroop Test to measure change in cognitive capacity following social interactions because of its use in previous studies, e.g. [49],[50]. However, since introversion has also been conceptualized as greater cortical sensitivity leading to a tendency to become overstimulated [65],[66], it may be that introverts feel increasingly uncomfortable in social situations because of overstimulation. This overstimulation may lead introverts to feel exhausted, but may not affect cognitive capacity. While the Stroop Test has been validated as a measure of cognitive capacity, it may not pick up on cognitive changes resulting from overstimulation. Furthermore, the relatively short period of social interaction may be another factor that explains why we did not observe an effect of extraversion on cognitive capacity after socializing. Although we attempted to develop a paradigm that captured what happens in real life, a 20-minute social interaction may not be tiring enough to influence performance on the Stroop Test. Future research should use other measures of cognitive capacity, e.g. working memory capacity [67], following a longer period of socializing in order to better test this hypothesis.

Another limitation of our study is that it was designed as an exploration of factors that might help explain why those lower in extraversion are less motivated to socialize, but was not designed to test specific mechanisms. The fact that those higher in extraversion are perceived as more socially skillful and this predicts greater increases in positive affect suggests that the relationship between extraversion and increases in positive affect may be mediated by higher quality social interactions, but we were unable to evaluate this possible explanation. Owing to our design, which measured post-interaction positive affect and perceived social skillfulness at the same time, it was not possible to determine whether being perceived as more socially skillful increases positive affect or whether increases in positive affect prompt heightened perceptions of social skillfulness. Previous research suggests that qualitative aspects of the social interaction mediate the link between extraversion and positive affect [38],[68]. For example, in an experience sampling study, social contribution mediated the relationship between natural fluctuations in extraverted behavior and positive affect in daily life [68], and in a longitudinal study, qualitative aspects of the social experience (i.e. sense of belonging and feeling socially connected), but not quantitative aspects (i.e. size of social network) mediated the relationship between extraversion at the beginning of college and subjective well-being four years later [38]. Thus, previous research suggests that social skillfulness may increase positive affect in a social interaction, but future research should delve further into exploring mechanisms underlying our effects.

Our sample included a predominance of women (70%) and the age range was restricted to those 18–30. Given this, our results may not generalize to men and to populations beyond this age range. A large study on age-related changes in Big Five domains and facets showed that extraversion levels do change over the lifecourse [69]. However, although extraversion decreases from late childhood into adolescence, extraversion trends are flat through adulthood [69]. Another limitation of our study design is that some participants were in groups with others that they already knew. Although participants were instructed not to interact with former acquaintances or respond to the questionnaire about them, future research should include a prescreening to ensure there are no pre-existing relationships among participants within a group. Finally, although participants rated each social interaction partner separately, they provided their general impression of how happy and energized they felt after all of the social interactions. Given that one social interaction may have been unpleasant, there may be memory biases particularly if this was their last social interaction.

### Conclusions

Taken together, our results suggest the following: the vast majority of people benefit hedonically from social interaction, as measured by their post-social interaction positive affect; however, those lowest in extraversion do not. Individuals lower in extraversion hold pessimistic expectations about these interactions, which may contribute to avoidance: they expect to feel worse, not better, after the interaction, and they harbor fears of being evaluated negatively. Paradoxically, though, those with the most fear about being negatively evaluated do better: their partners find them more skilled than their low-extraversion counterparts who do not share this fear of negative evaluation. Given that these findings came from a single exploratory study, our findings should be interpreted as preliminary and should be replicated. In addition, future research should uncover the specific mechanisms that lead individuals lower in extraversion to harbor such pessimistic predictions about their experiences in social interactions and the precise mechanisms underlying their diminished positive affect boost following social interaction.
